# TGV Upsampling: A Making-Up Operation for Semantic Segmentation

**DOI:** 10.1155/2019/8527819

**Published:** 2019-08-01

**Authors:** Xu Yin, Yan Li, Byeong-Seok Shin

**Affiliations:** Department of Computer Engineering, Inha University, Incheon. 082, Republic of Korea

## Abstract

With the widespread use of deep learning methods, semantic segmentation has achieved great improvements in recent years. However, many researchers have pointed out that with multiple uses of convolution and pooling operations, great information loss would occur in the extraction processes. To solve this problem, various operations or network architectures have been suggested to make up for the loss of information. We observed a trend in many studies to design a network as a symmetric type, with both parts representing the “encoding” and “decoding” stages. By “upsampling” operations in the “decoding” stage, feature maps are constructed in a certain way that would more or less make up for the losses in previous layers. In this paper, we focus on upsampling operations, make a detailed analysis, and compare current methods used in several famous neural networks. We also combine the knowledge on image restoration and design a new upsampled layer (or operation) named the TGV upsampling algorithm. We successfully replaced upsampling layers in the previous research with our new method. We found that our model can better preserve detailed textures and edges of feature maps and can, on average, achieve 1.4–2.3% improved accuracy compared to the original models.

## 1. Introduction

Compared to traditional classification tasks, semantic segmentation is much more difficult. From the level of the neural network, a classifier should be used for each pixel of an image. This can help machines have a better understanding of complex images, not only simple object recognition. Deep learning methods, especially convolutional neural networks, have generated spectacular results for visual recognition problems. This has proven that convolutional operations can successfully extract global information and features for maintaining spatial invariances. When looking back on existing studies, we observed that most of them follow a symmetric architecture, which decreases the resolution (called the encoding stage) and then gradually increases it layer by layer (called the decoding stage). Whatever detailed designs are, for feature maps, if a network contains a high-to-low process, it will bring inevitable losses because this process aims to generate a more liable low-resolution presentation from a high-resolution one. Meanwhile, with a layer increased, there would be a great effect on the texture (or boundary information) of the final dense images.

One way to handle this challenge is to reduce the loss of operations during performance. Chen et al. [1] used an atrous convolution. By inserting “blanks” between pixels, the researchers enlarged the receptive field and generated a higher resolution feature map. Although this method is effective and successful, it is still hard to apply, considering the limitations of hardware and memory.

Another solution focuses on the methods of making up for losses. In recent research, a design of hierarchical networks [2, 3] has been a trend. Researchers found that losses can be made up for in later stages. High-level feature maps can be better recovered when mixing information of low-level intermediate results, which further improves dense predictions.

**Figure 1:**
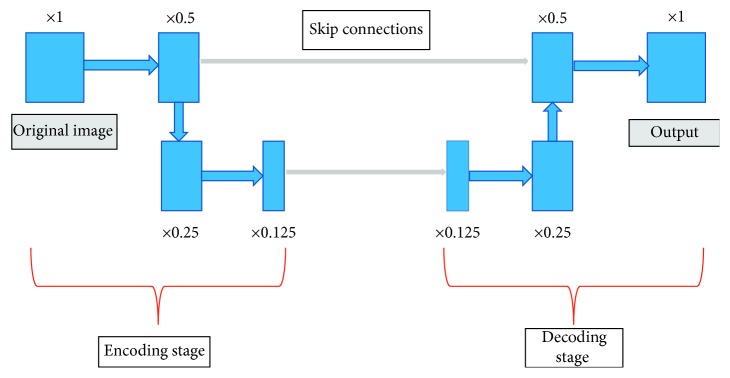
General symmetric network architecture.

We were inspired by the making-up idea and trend to group the layers into encoding and decoding stages (see Figure 1). In the encoding stage, layers downsample the spatial resolution layer by layer; afterward, in the decoding stage, feature maps (intermediate results produced by layers) are “upsampled” and resolutions are increased correspondingly until the entire image is reconstructed to the original size. Researchers believe that, in the down-up sampling process, in which images are segmented and reconstructed, networks can detect the most important features without destroying the shapes or textures of objects. The aim of reconstruction is to make up for the losses produced in the encoding stage, and the choice of the upsampling method is the key point.

In this paper, we follow symmetric designs and focus on the research of upsampling methods in the decoding stage. Our contributions are as follows:

1. We made a detailed introduction of upsampling methods that are now commonly used, necessary concepts, and mathematical definitions.
2. We proposed a new upsampling method based on the total generalized variation (TGV) model [4–6] and applied it to different networks.

## 2. Related Works

Semantic segmentation, an important branch of deep learning [7], has also become an active topic in neural network research. In this section, we introduce several advanced networks in the field of semantic segmentation and their upsampling methods used in the decoding stage.

### 2.1. Symmetric-Like Networks

Symmetric networks [8–11] have been proven to be highly effective in particular fields. As for the reason for choosing a symmetrical type, researchers thought that regardless of the convolutional layer, there would be a loss of information when extracting features. With further training, even a small loss may result in defects in boundary information or texture edges. To make up for the loss, they grouped all layers in a model into two stages according to the size of the feature maps. Long et al. [9] first used a fully convolutional network (FCN). They advised that fully connected layers can be replaced by convolutional layers, and further improved connections can be made between stages via “skip connections.” Although the FCN has incomplete symmetry, a skip connection, which builds a “bridge” between layers in the encoding stage and decoding stage, provides another source for a feature map. Based on this idea, Ronneberger et al. [8] designed a U-shaped network (U-Net). They considered that the expansive path of features has a relationship with the contracting path and thus enlarged the number of feature channels. However, when we want to design a new network, parameter size is a significant problem that much importance should be attached to, considering that symmetric networks need many more parameters than other kinds of networks, which would be a great challenge to CPU and GPU usage. At almost the same time, Badrinarayanan et al. [10] applied a new upsampling method named “unpooling,” which records the indices when max-pooling. With these indices, original location information can be easily found in the decoding stage. In this case, parameters needed in training processes are greatly reduced.

### 2.2. Current Upsampling Methods

With the visualization of convolutional networks [12], we know that convolutional computations can effectively extract and generalize features, outputs of which can be seen as a set of features. The goal of upsampling operations is to increase the resolution from low-resolution map data and to upsample original data to a high-resolution map, aiming to make up for the information lost in previous layers because of convolution operations.

The difficulty of the entire process lies in how to generate sampling data from low-resolution maps and corresponding colour channels.

#### 2.2.1. Bilinear Interpolation

In early deep learning research [7, 13], this kind of method could often be seen. Compared to methods introduced later on, the greatest advantages of the bilinear method are high speed and simple operations. From the level of layers, there is no need to learn and adjust weights when using bilinear interpolation, as one parameter (referring to the desired size of the final image) is enough for the entire operation.

For example, with four known point values, *x*_11_=(*x*_1_, *y*_1_), *x*_12_=(*x*_1_, *y*_2_), *x*_21_=(*x*_2_, *y*_1_), and *x*_22_=(*x*_2_, *y*_2_), the value of function *τ* can be found at the point (*x*, *y*).

*X*-direction:

(1) $$\begin{array}{c} Q_{\left(x,y_{1}\right)}=\tau \left(x,y_{1}\right)\approx \frac{x_{2}-x}{x_{2}-x_{1}}\tau \left(x_{11}\right)+\frac{x-x_{1}}{x_{2}-x_{1}}\tau \left(x_{21}\right),\\ Q_{\left(x,y_{2}\right)}=\tau \left(x,y_{2}\right)\approx \frac{x_{2}-x}{x_{2}-x_{1}}\tau \left(x_{12}\right)+\frac{x-x_{1}}{x_{2}-x_{1}}\tau \left(x_{22}\right). \end{array}$$

*Y*-direction (yielded):

(2) $$\begin{array}{c} Q_{\left(x,y\right)}=\tau \left(x,y\right)\approx \frac{y_{2}-y}{y_{2}-y_{1}}Q_{\left(x,y_{1}\right)}+\frac{y-y_{1}}{y_{2}-y_{1}}Q_{\left(x,y_{2}\right)}\\ =\frac{\tau \left(x_{11}\right)}{\left(x_{2}-x_{1}\right)\left(y_{2}-y_{1}\right)}\left(x_{2}-x\right)\left(y_{2}-y\right)+\frac{\tau \left(x_{21}\right)}{\left(x_{2}-x_{1}\right)\left(y_{2}-y_{1}\right)}\left(x-x_{1}\right)\left(y_{2}-y\right)+\frac{\tau \left(x_{21}\right)}{\left(x_{2}-x_{1}\right)\left(y_{2}-y_{1}\right)}\left(x_{2}-x\right)\left(y-y_{1}\right)+\frac{\tau \left(x_{22}\right)}{\left(x_{2}-x_{1}\right)\left(y_{2}-y_{1}\right)}\left(x-x_{1}\right)\left(y-y_{1}\right). \end{array}$$

With the above processes, it is possible to find an approximation value of any point (*x*, *y*) within the function interval (*x* ∈ (*x*_1_, *x*_2_), *y* ∈ (*y*_1_, *y*_2_)).

#### 2.2.2. Transposed Convolution

Strictly speaking, transposed convolution is a kind of convolution operation instead of a sampling method, but many researchers are currently making it their first choice to make up for losses in the decoding stage.

From the perspective of convolution arithmetic, each convolution operation can be represented as *y*=*C* *∗* *x*, where *C* and *x* stand for weight matrix and input maps, respectively.

In the period of backpropagation, assuming that the derivative of layers ∂Loss/∂*y* (Loss means the loss of the whole network when training) is known, corresponding derivatives of weights can be written as

(3) $$\begin{array}{c} \frac{\partial \text{Loss}}{\partial x}=\underset{i}{\sum }\frac{\partial \text{Loss}}{\partial x_{i}}=\underset{i}{\sum }\frac{\partial \text{Loss}}{\partial y_{i}}\;*\;\frac{\partial y_{i}}{\partial x_{j}}\\ =\underset{i}{\sum }\frac{\partial \text{Loss}}{\partial y_{i}}\;*\;C_{ij}=\frac{\partial \text{Loss}}{\partial y}\;*\;C_{*j}\\ =C_{*,j}^{T}\;*\;\frac{\partial \text{Loss}}{\partial y}. \end{array}$$

Equation (3) shows the relationship of ∂Loss/∂*x* and ∂Loss/∂*y*. It shows that transposed convolution is actually multiplying *C* ^*T*^ or (*C* ^*T*^)^*T*^=*C* when doing a forward pass and a backward pass, respectively.

When transposed convolution is used as the upsampling method, situations are totally different from bilinear interpolation. The study of Dumoulin and Visin [14] introduced four different cases where a transposed operation was applied.

**Figure 2:**
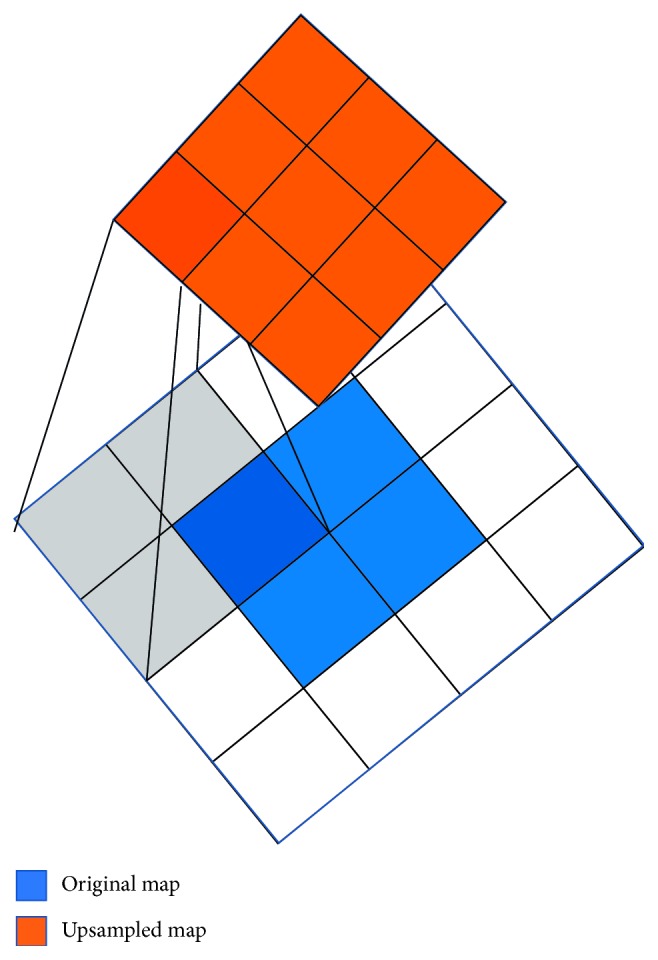
Transposed convolution.

For instance (Figure 2), the transposed convolution over a 2 × 2 input for a 3 × 3 output (stride = 1) equals a reverse operation of the convolutional layer (*y*=*C* *∗* *x*, with input 4 × 4; kernel size = 2). The original 2 × 2 map is padded with a 2 × 2 border of zeros first, and then *C* ^*T*^ is multiplied.

#### 2.2.3. Unpooling

Various combinations of layers have currently improved the efficiency of networks, but they brought a severe problem at the same time: parameters. Even a single convolutional layer requires a high number of weight parameters, which poses a significant challenge for CPUs or GPUs. As noted in Section 2, transposed convolution equals multiplying a transposed matrix of corresponding convolutional layers, meaning that we should spare enough memory to save these matrices when training.

First utilized in deconvolutional networks [15], unpooling is much simpler and easier to use.

**Figure 3:**
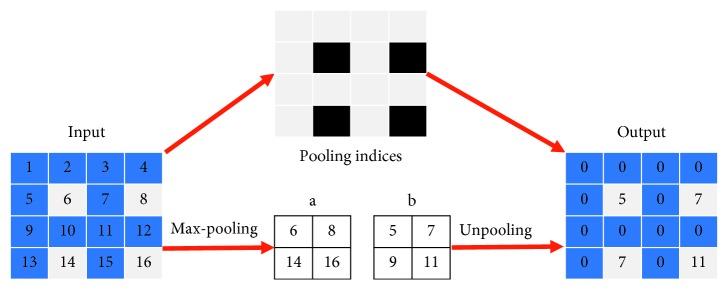
Unpooling operation.

Figure 3 shows a basic process of an unpooling operation. All numbers above correspond to values in a feature map. Assume that for some map input the unpooling method records indices of the largest values before performing max-pooling (from input ⟶ *a*). In the decoding stage, having received outputs *b* from previous layers, with pooling indices (represented by the black grids), it is possible to upsample the pixel values inside to their original places (from *b* ⟶ output). In fact, unpooling keeps the directional information (“pooling indices”) of the largest pixel values in each feature map when processing max-pooling operations. This action solves the problems of “directions” and “padding” at the same time.

Compared to transposed convolution, the advantage of unpooling lies in the number of parameters, which should only record the pooling indices.

## 3. TGV Upsampling Algorithm

Inspired by the total variation model [16, 17], we introduce another upsampling method named “TGV upsampling.” We see upsampling methods as a way to combine feature map restoration with knowledge of image restoration (with the aim of making up for loss of information). The background and details of our method are described as follows.

### 3.1. Problem Transformation

Although it has been several years since the first convolutional neural network (CNN) was used [7], the internal architecture can still be simply concluded. Most networks now can be thought of as a combination of convolutional layers and max-pooling layers, and there are a lot of research studies [14, 18–21] available for understanding what CNN is. Many previous studies pointed out that “max-pooling can be replaced by a convolutional layer with increased stride and without loss in accuracy at the same time” [22]. Certainly, we can think of network components with max-pooling and convolutional layers as a set of convolutional computations regardless of what the internal architecture actually is.

As we discussed in previous sections, in the field of semantic segmentations, researchers have tended to divide the whole process into encoding and decoding stages where the upsampling step always happens in the decoding stage. Many researchers thought that a “path” exists in convolution operations. From the perspective of feature maps, stages of encoding and decoding refer to the expanding and contracting paths, respectively; these two paths are more or less symmetrical. Under such circumstances, we treat upsampling as a restoration task of feature maps produced from corresponding layers in encoding stages.

For example, Figure 1 provides a simple view of a general symmetric-like network. The whole network is grouped into encoding and decoding stages, and layers in the two stages are represented by *E*_*i*_ and *C*_*j*_, respectively (where *i* and *j* stand for the direction of the layer). When *i* = *j*, it means *E*_input_ = *C*_output_, and we consider the outputs of *C*_*j*_ as the result of feature map restoration to *E*_input_.

**Figure 4:**
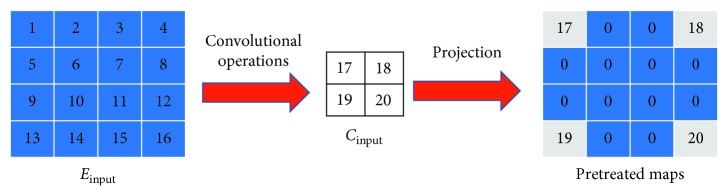
Pretreatment to feature maps.

As shown in Figure 4, after a set of convolutional computations, a 4 × 4 feature map in the encoding stage becomes a 2 × 2 pixel matrix (this matrix is also the output of a certain layer). With a projection operation, this map can be recovered to the original size of the map in the encoding stage (this temporary result is denoted by ${\bar{C}}_{\text{input}}$). The padding part (referring to the heavy blue grids) appears as blurred (noisy) areas; the rest of the work is a restoration job.

When comparing the left matrix with the right one, we formulate the basic loss function as follows:

(4) $$\begin{array}{c} \text{loss}\left(u,C_{\text{input}}\right)=\frac{1}{2}{\left\|u\left(C_{\text{input}}\right)-{\bar{C}}_{\text{input}}\right\|}_{2}^{2}, \end{array}$$

where *u* stands for our upsampled result and ‖·‖_2_ denotes 2-norm.

### 3.2. TGV-Based Upsampling

TGV [23] is mainly used to solve problems like image denoising and restoration. But in this paper, we adapted it into a trainable upsampling method of feature maps. We increase the resolution of maps to a target size and then perform TGV on each map.

The first step in our method is projection, considering the size difference between low- and high-resolution maps. We first applied bilinear interpolation to get a preliminary result, which has the same size as maps in the encoding stage. These processed maps will be seen as noisy areas (actually referring to the entire image), which would be treated in TGV restoration.

Since Bredies et al. [23] put forward the TGV model, it has been widely used in the field of image restoration. The TGV model greatly reconstructs an image from blurred data or noisy, indirect measurements. We formulated the whole upsampling problem as a convex optimization model [12, 24–26]. The mathematical formulations for our model are outlined as

(5) $$\begin{array}{c} \underset{u}{\min}\left\{\lambda \text{loss}\left(u,x\right)+{\text{TGV}}_{\alpha }^{K}\left(u\right)\right\}, \end{array}$$

where loss(*u*, *x*) represents image fidelity, the parameter *λ* is used to weight previous operations to compute a global optimization, and TGV_*α*_^*K*^(*u*) stands for the regularization term.

With the visualization of convolutional networks [8], we know that convolutional computations can effectively extract and generalize features, outputs of which can be seen as a set of features. To better understand the semantics of an image, smoothness of textures and border edges are particularly important to feature maps.

For a *k*-order image, traditional restoration methods tend to fix the bounded variation seminorm, whereas the TGV model introduces a *k*-order function and incorporates information from different channels, which can effectively maintain texture discontinuities.

Given a *k*-order image *μ*, we represent the TGV model as follows:

(6) $$\begin{array}{c} {\text{TGV}}_{a}^{k}\left(u\right)=\sup\left\{\int_{\Omega }\Omega div^{k}\;v\;dx\;|\;v\in C_{C}^{K}\left(\Omega ,{\text{Sym}}^{k}\left({\text{IR}}^{d}\right)\right),\left\|div^{l}\;v\right\|\leq a_{l},\;l=0,\ldots ,k-1\right\}. \end{array}$$

Let Sym^*k*^(IR^*d*^) represent the space of symmetric tensors of order *k*. By balancing derivatives from the first order to the *k*th order, it greatly alleviates the problem of containing different grey levels when imaging, leading to boundary information loss as well as edge and corner loss.

But in this work, aiming at feature maps, it turns out that a 2-order TGV is sufficient. We formulate the 2-order model as

(7) $$\begin{array}{c} {\text{TGV}}_{a}^{2}\left(u\right)=\underset{w}{\min}\left\{a_{1}\int_{\Omega }\left|\nabla u-\omega \right|+a_{2}\int_{\Omega }\left|\varepsilon \left(\omega \right)\right|\right\}. \end{array}$$

For a given 2-order *u*, the minimum of the TGV model is taken over all complex vector fields *w* in the bounded domain. *ε*(*w*)=1/2(∇*ω*+∇*ω* ^*t*^) stands for the symmetrized derivative. Parameters *a*_1_ and *a*_2_ are applied to balance the first and second derivatives.

The final TGV-based upsampling model is defined as a combination of the loss function (5) and the TGV term (6) as equation (7):

(8) $$\begin{array}{c} \underset{u,w}{\min}\left\{\frac{\lambda }{2}{\left\|u\left(C_{\text{input}}\right)-{\bar{C}}_{\text{input}}\right\|}_{2}^{2}+a_{1}\int_{\Omega }\left|\nabla u-\omega \right|+a_{0}\int_{\Omega }\left|\varepsilon \left(\omega \right)\right|\right\}. \end{array}$$

Meanwhile, unlike fixed weights used in [23], we adapt TGV into a trainable method, taking weights a_1 and a_2 into backpropagation to search for a suitable balance point.

### 3.3. Optimization Methods

Considering that the upsampling model we proposed (formula (8)) is convex and not smooth, we used the primal-dual scheme [24, 25, 27] to solve this problem. We reformulated our upsampling model as a convex-concave saddle-point problem by introducing two dual variables *m* and *n*. The transformed model of formula (8) is provided by

(9) $$\begin{array}{c} \underset{u}{\min}\underset{v}{\max}\frac{\lambda }{2}\underset{i,j\in \Omega }{\sum }{\left(u\left(C_{i,j}\right)-E_{i,j}\right)}^{2}+a_{1}\langle \left(\nabla u-\omega \right),m\rangle +a_{2}\langle \nabla \omega ,n\rangle . \end{array}$$

The feasible sets of *ρ* and *φ* are defined as follows:

(10) $$\begin{array}{c} M=\left\{m:\Omega_{u}⟶{ℛ}^{2}\;|\;\left\|m_{i,j}\right\|\leq 1,i,j\in \Omega_{u}\right\},\\ N=\left\{n:\Omega_{u}⟶{ℛ}^{2}\;|\;\left\|n_{i,j}\right\|\leq 1,i,j\in \Omega_{u}\right\}. \end{array}$$

The final result is computed pixel by pixel via iterative optimization. With *u* ^0^=*E*_input_ and *v* ^0^, *m* ^0^, and  *n* ^0^=0, step sizes *σ*_*m*_, *σ*_*n*_ and *τ*_*u*_, *τ*_*ω*_ are chosen. For iteration *i* ≥ 0, updated variables are as follows:

(11) $$\begin{array}{c} \left\{\begin{array}{c} m^{i+1}={\text{Proj}}_{m}\left\{m^{i}+\sigma_{m}a_{1}\left(\nabla {\tilde{u}}^{i}-{\tilde{\omega }}^{i}\right)\right\},\\ n^{i+1}={\text{Proj}}_{n}\left\{n^{i}+\sigma_{n}a_{2}\nabla {\tilde{\omega }}^{i}\right\},\\ u^{i+1}={\left(1+\frac{\lambda }{2}\tau_{u}\right)}^{-1}\left[u^{i}+\tau_{u}\left(a_{1}m^{i+1}+\frac{\lambda }{2}{\bar{C}}_{\text{input}}\right)\right]\\ \omega^{i+1}=\omega^{i}+\tau_{\omega }\left(a_{1}n^{i+1}+a_{2}m^{i+1}\right),\\ {\bar{u}}^{i+1}=u^{i+1}+\theta \left(u^{i+1}-{\bar{u}}^{i}\right),\\ {\bar{\omega }}^{i+1}=\omega^{i+1}+\theta \left(\omega^{i+1}-{\bar{\omega }}^{i}\right), \end{array}\right., \end{array}$$

where ${\text{Proj}}_{m}\left(\bar{m}\right)$ and ${\text{Proj}}_{n}\left(\bar{n}\right)$ denote the Euclidean projectors onto the sets *M* and *N*, respectively. They can be calculated by pointwise operations:

(12) $$\begin{array}{c} {\text{Proj}}_{m}\left(\bar{m}\right)=\frac{\bar{m}}{\max\left(1,\left|\bar{m}\right|/a_{1}\right)},\\ {\text{Proj}}_{n}\left(\bar{n}\right)=\frac{\bar{n}}{\max\left(1,\left|\bar{n}\right|/a_{1}\right)}. \end{array}$$

In equation (11), *θ* stands for the relaxation parameter. It can be updated iteration by iteration using preconditioning [5]. Hence, the TGV-based model can achieve globally optimal upsampling solutions when dealing with 2-order images.

## 4. Evaluation

In this section, we make a quantitative evaluation of the proposed TGV-based upsampling model. We compared our model with current upsampling methods from different perspectives. We used a PASCAL VOC2012 dataset (class segmentation) to investigate the detailed advanced performances. In the following experiments, we manually set the TGV-based upsampling model's parameters *a*_1_ and *a*_2_ (in our experiments, we initially set *a*_1_=0.05 and *a*_2_=0.1; both of them can be trained with iterations).

We retrained original networks (FCN, U-Net, and SegNet) and their modified versions in the TGV upsampling algorithm on a PASCAL training set (with 1464 images).

**Figure 5:**
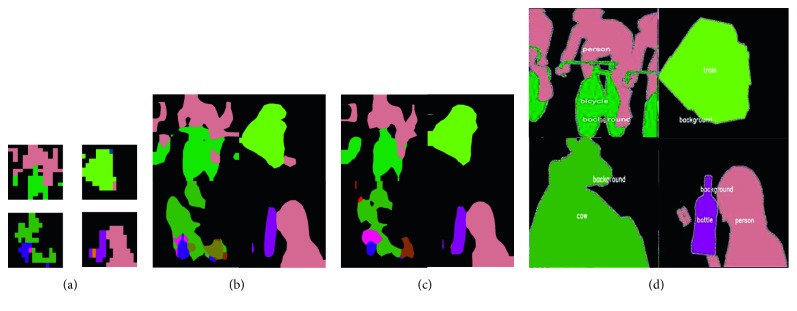
Comparison between a transposed operation and TGV upsampling for the same feature maps (in FCN). (a) Input maps. (b) TGV upsample. (c) Transposed. (d) Ground truth.

**Figure 6 fig6:**
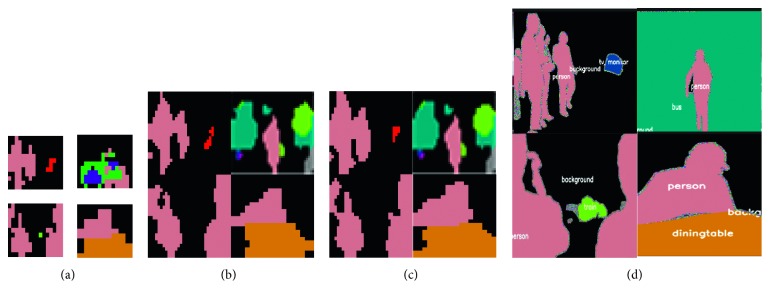
Comparison between a bilinear operation and TGV upsampling for the same feature maps (in U-Net). (a) Input maps. (b) TGV upsample. (c) Bilinear. (d) Ground truth.

**Figure 7:**
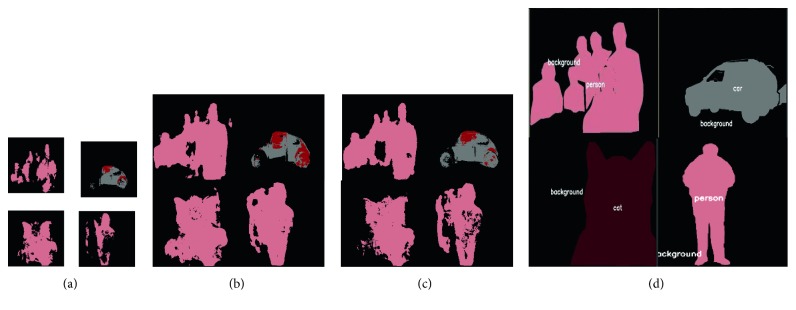
Comparison between an unpooling operation and TGV upsampling for the same feature maps (in SegNet). (a) Input maps. (b) TGV upsample. (c) Unpooling. (d) Ground truth.

For a reconstruction comparison on feature maps (Figures 5 [Fig fig6]–7), we evaluated the above four methods on a PASCAL validation set (with 1449 images). We randomly select feature maps from a certain layer in FCN, U-Net, and SegNet and compared the upsampled results of using different methods.

**Figure 8:**
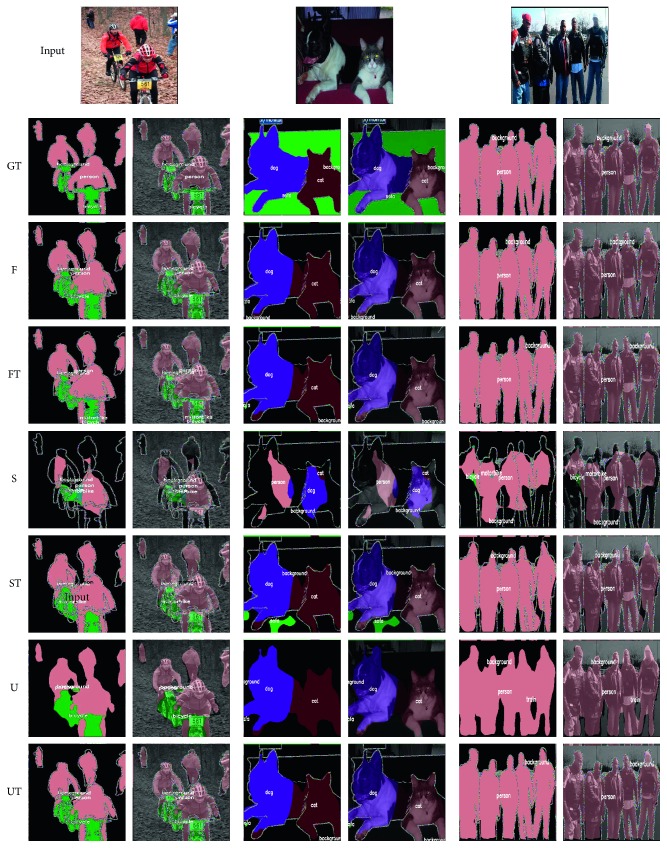
Overall segmentation example results on PASCAL VOC (GT stands for ground truth, F, S, and U represent results from FCN, SegNet and U-Net, while FT, ST, and UT mean three networks combined with TGV upsampling).

For overall comparisons of segmentation results (Figure 8), we used a PASCAL test set (with 1456 images). For quantitative results (with 1456 images), we adopted overall accuracy (OA), mean accuracy (MAcc), frequency-weighted intersection over union (FWIoU), and mean intersection over union (MIoU) as metrics.

### 4.1. Experiments

#### 4.1.1. Reconstruction of Intermediate Feature Maps

**Figure 9:**
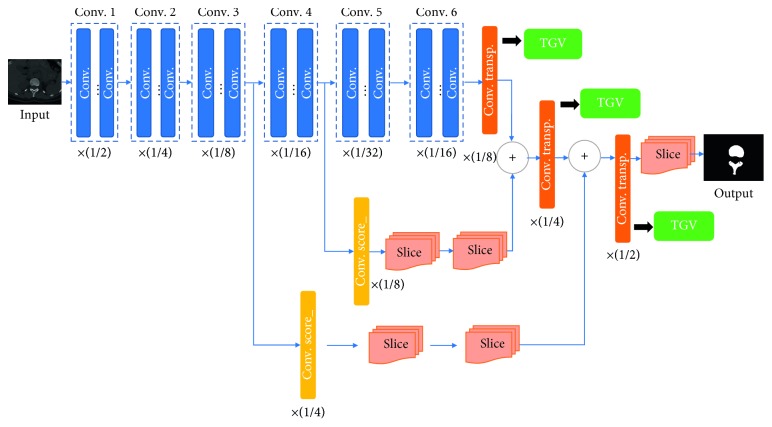
Replacing the transposed convolutional layer in the FCN with the TGV upsampling method.

In this stage, we replaced the original upsampling layer in all three networks (FCN, U-Net, and SegNet) with our TGV upsampling method (see Figure 9, with FCN as an example). To better compare results, we illustrated the performance of TGV upsampling when dealing with the same inputs (feature maps produced by previous layers) and so on, replacing bilinear operations in U-Net and unpooling in SegNet.

Considering that certain upsampling methods may multiply when used in models, we only selected one comparison regardless of the size of the inputs and how many upsampling operations appeared in the networks. Feature maps in Figures 5 [Fig fig6]–7 are the most representative of activation outputs in each layer in the training process.

In Figures 5 [Fig fig6]–7 (illustrating the comparisons of the feature maps), all input maps are from some layer in the encoding stage. We found that TGV upsampling performed better at saving the textures and edges of the feature map. In Figure 5, with the same input maps from the FCN architecture, we observed that compared with transposed convolution reconstruction, TGV upsampling reproduced almost all classes (represented in different colors) that appeared in input maps. While being a trainable operation, the transposed operation processes inputs following a “padding and extracting” pipeline. There absolutely exists a certain chance that sampled maps contain some classes that in fact are contained in input maps, or a class may be filtered. It can be clearly seen that, in the second and third input submaps (Figure 5), the transposed operation did not completely reproduce all objects appearing in the input map. Even if these cases are ignored, it is easy to observe that TGV upsampling is better in reconstructing texture than transposed operations.

As illustrated in Figure 6, bilinear interpolation recovered too much unnecessary information about known classes from both *x* and *y* directions of the object, compared to the principle of TGV upsampling reconstruction, based on the high-order divergence of the known pixel values. The source of the TGV model's information was more reasonable and more complex. The reconstruction results of TGV upsampling were also better than those of bilinear interpolation.

Lastly, Figure 7 compares unpooling and TGV upsampling. Since unpooling only records the locations of maximum activations when performing pooling operations in the encoding stage, it is clear that the unpooling reconstruction is not complete, as there are many blank spaces in places where texture should appear. In general, the efficiency of the unpooling operation is the worst of the three in terms of visualization results.

#### 4.1.2. Training in TGV Upsampling

In this experiment, we compared overall segmentation results with those of the original networks.

Figure 8 illustrates examples of PASCAL VOC outputs from FCN (FCN-8s), U-Net, SegNet, and the modified TGV upsampling-based networks (denoted by FCN-TGV, U-Net-TGV, and SegNet-TGV, respectively). Compared to the coarse-feature maps of the original networks, class (or object) recognitions (referring to the interval area between objects) in our results are more precious (see SegNet and SegNet-TGV) and the situations of exceeding areas in ground truth are greatly eased (see FCN and FCN-TGV). At the same time, they well preserved the smoothness of the boundary. Lastly, it can be concluded that the TGV upsampling method helps models make a better predication on semantics.

**Figure 10:**
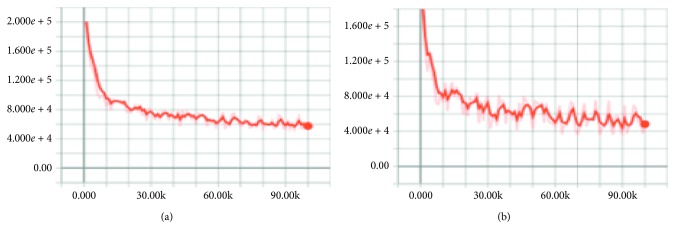
Loss function of SegNet (a) and SegNet-TGV (b) on the PASCAL VOC VALIDATAE set.

The quantitative results of the VOC test set of our proposed algorithm and the competitors are presented in Table 1. We observed that, in terms of overall accuracy, mean accuracy, frequency accuracy, or MIoU, all metrics were improved by about 1.4–2.3%, corresponding to the visualization results in Figure 10.

**Figure 11 fig11:**
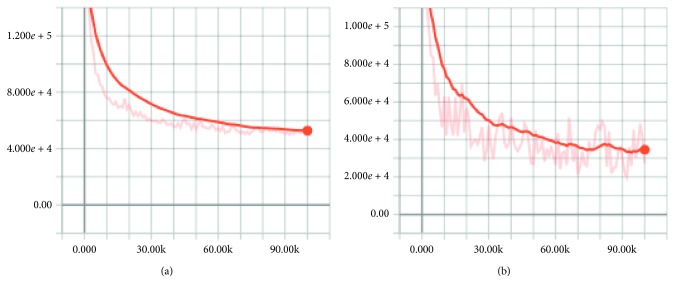
Loss function of FCN (a) and FCN-TGV (b) on the PASCAL VOC VALIDATAE set.

**Figure 12:**
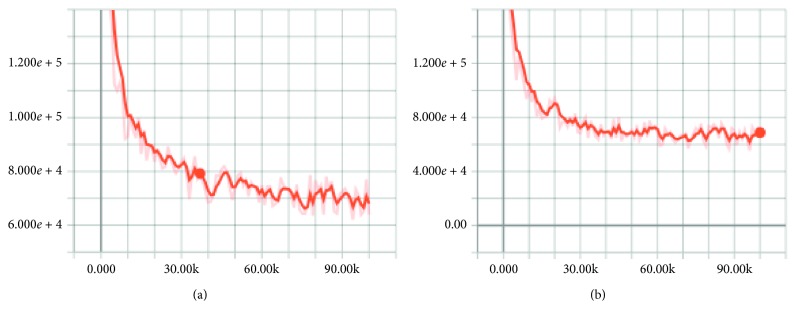
Loss function of U-Net (a) and U-Net-TGV (b) on the PASCAL VOC VALIDATAE set.

Figures 10 [Fig fig11]–12 show the effect after applying the new upsampling method (the *X* and *Y* axes represent iterations and losses, respectively). We found that the TGV upsampling algorithm had a significant effect on reducing losses compared to the original methods. With a fixed batch size, our new method converged faster than the original models.

#### 4.1.3. Loss Function in the Training Process

The loss function of FCN, FCN-TGV, U-Net, U-Net-TGV, SegNet, and SegNet-TGV on the PASCAL VOC VALIDATAE set is given in Figures 10 [Fig fig11]–12.

### 4.2. Analysis

After concluding all four upsampling operations introduced in the above sections, we can group them into two types based on their different information sources.

#### 4.2.1. Back-Up (Including Unpooling and Transposed Convolution Operations)

Many researchers think that when reconstructing feature maps, intermediate results produced in previous operations should be referred to (especially layers in the encoding stage) as if images are processed along a “path.” Having known conditions of front distances (referring to corresponding layers in the encoding stage) makes it possible to better “predicate” the following road conditions (referring to feature map reconstructions). Thus, when applying these methods, operation information of previous layers should be saved. For example, the unpooling operation is actually seen as an index storage. With saved parameters, feature maps can be well reconstructed.

However, in both transposed and unpooling operations, it is unavoidable that the processed results would retain a certain part of the intermediate results produced by previous layers. In some cases, this is useful for keeping important information that may be overlooked in the training process, but it can also be a disturbance by bringing along unnecessary information.

#### 4.2.2. From-Itself (Including Bilinear Interpolation and Our TGV Upsampling Operations)

In contrast to the “back-up” category, the information sources bilinear interpolation and TGV upsampling used to make up for losses are coming from the methods' processes rather than results of previous operations. As described in Related Works, bilinear operation interpolates from *x* and *y* directions based on known sample values. The TGV method has a more complex information source. It starts from *k*-order divergence of known pixel values (for *k*-order images). It can better preserve boundary information of textures, and the step effect can be effectively avoided, raising the smoothness of the entire image at the same time.

From the results of these experiments, the numerical results of the loss function and the smoothness of the sampled feature maps have proven that the TGV-upsampling method is a great improvement over current methods.

## 5. Conclusions

In this work, we introduced a TGV-upsampling method based on image restoration research. We transformed the process of feature map reconstruction into a loss-optimization problem. Based on the divergence of each order, we used TGV regularization, which reconstructs piecewise functions. For numerical optimization, we used a primal-dual algorithm, which can effectively perform parallel computing and result in high frame rates. We applied our new method to three networks and evaluated the results via PASCAL VOC datasets. These tests proved that the TGV upsampling method can greatly make up for lost smoothness and boundary information of maps. Compared to the original methods used in networks (FCN, U-Net, and SegNet), this new method made average improvements of 1.4–2.3% in terms of MIoU accuracy when used in the decoding stage. When observing the training process in the experiments, we clearly found that the TGV upsampling method greatly made up for information losses that resulted from other operations (mainly referring to various convolutional layers).

The proposed algorithm is not limited to single-layer map upsampling. In the future, it will be extended to understanding entire scenes for effectively incorporating temporal coherence in a consistent way. On the contrary, with the proposed “restoration-like” method, we will further concentrate on how to better understand latent semantics under unsupervised settings.

## Figures and Tables

**Table 1:** Results of the PASCAL VOC set.

Method	OA	MAcc	FWIoU	MIoU
FCN-8s	91.13	78.68	84.59	64.59
**FCN-TGV**	92.33	80.12	87.49	66.02
SegNet	86.86	74.41	80.32	58.59
**SegNet-TGV**	87.46	76.08	83.45	60.48
U-Net	87.13	70.68	79.59	61.59
**U-Net-TGV**	89.14	72.12	81.49	63.89

## Data Availability

The codes used to support the findings of this study have not been made available because the proposed method would later be applied in medical projects that involve personal privacy.
